# Phenotypic correlations in a patient with ring chromosome 22

**DOI:** 10.4103/0971-6866.69372

**Published:** 2010

**Authors:** Osman Demirhan, Erdal Tunç

**Affiliations:** Department of Medical Biology and Genetics, Faculty of Medicine, University of Çukurova, Adana, Turkey

**Keywords:** Dysmorphic features, mental retardation, ring chromosome 22

## Abstract

Ring chromosome 22, a rare cytogenetic anomaly, has been described in over 60 cases in the medical literature. The aim of this report was to present a case carrying ring chromosome 22, and her family.

It is a case report of a patient presented at Medical Faculty of Çukurova University in Turkey.

An 8-year-old girl with ring chromosome 22 and her family were evaluated cytogenetically and clinically.

A chromosome analysis of the proband revealed a *de novo* 46, XX, r(22)(p11.2;q13) karyotype. Our subject demonstrated the prominent features of this syndrome including profound mental retardation, language impairment, dysmorphic features, lack of speech, hyperactivity, and behavioral disorders.

There is lack of consistency between the physical abnormalities that we observed in our subject and those observed for such patients in the literature. The wide range of manifestations observed in patients with this cytogenetic alteration is probably due to size differences in the deleted region.

## Introduction

Ring chromosomes are formed by breakage in both arms of a chromosome with fusion of the points of fracture and loss of the distal fragments. Ring chromosome 22, a rare cytogenetic anomaly, was firstly described by Weleber *et al*. in 1968.[1] Since then, approximately 60 cases have been described in the medical literature. Its most consistent findings are overall developmental delay with severe speech disability, growth retardation with frequently associated microcephaly, hypotonia, and dysmorphic traits such as epicanthus, mental retardation, normally placed but large and dysplastic ears, long face, thick eyebrows, 2–3 toe syndactyly, long eyelashes with full eyebrows, and occasionally high arched palate, dental malocclusion, and mild hypertelorism.[1,2] Second and third toe syndactyly, unsteady gait, hyperactivity, aggressive behavior, autistic disorders, seizures, and abnormal EEG1 have also been reported.[3] These manifestations suggest that the central nervous system development of the affected patients is also altered. In addition to ring formation, deletion of the 22q13 region of chromosome 22 represents a recognizable cytogenetic microdeletion syndrome. There are currently 23 such cases published in literature,[4,5] and the clinical characteristics present in all patients include severely retarded speech, moderate mental retardation, and hypotonia. The clinical variations observed can result from the occurrence of different sized deletions. Molecular studies of the region[5] have revealed both the size of the critical region and the function of some of its genes.[6]

We report the cytogenetic analysis of ring chromosome 22 in a girl investigated for mental retardation, minor dysmorphic features, severe speech delay, hyperactivity, and aggressive behaviors.

## Case Report

**Figure 1:**
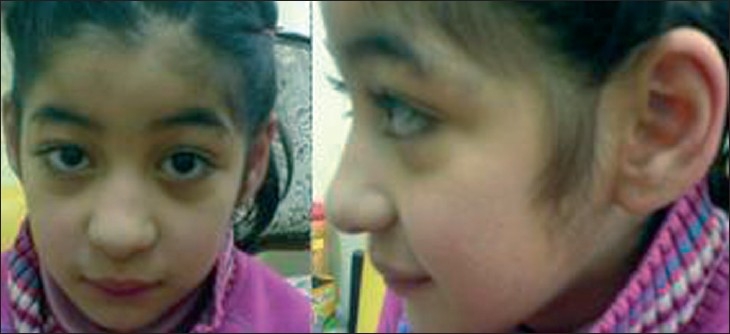
Frontal and lateral views of the patient at 8 years showing frontal hair upsweep, long face, low-set ears, thick eyebrows, long eyelashes, epicanthic folds and full eyebrows

**Figure 2:**
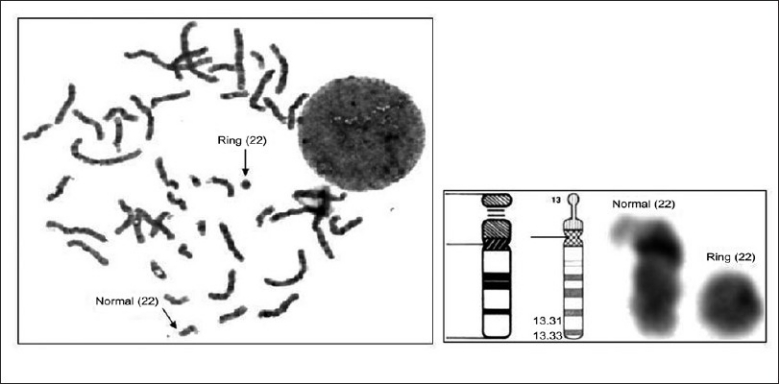
Karyotype documenting ring 22

The proband, an 8-year-old girl, was the second child born to healthy non-consanguineous parents, after normal gestation. There was no family history of intellectual sation handicap or mental illness. She was diagnosed with mental retardation at 3 years of age. She was referred to the Child Disease Department because of the complaints of mental retardation and speech delay. She had no complications at birth and her psyschomotor milestones were always behind her peers. She also had epicanthus, normally placed but large and low-set ears, long face, thick eyebrows, long eyelashes with full eyebrows, epicanthic folds, hyperactivity, and aggressive behavior [Figure 1]. Magnetic resonance imaging of the brain showed dysmyelini at 8 years of age. Also, the patient was showing significant behavioral changes, behavioral deterioration, and her speech was meaningless. Chromosome analysis of the proband, using cultured lymphocytes and giemsa banding technique showed a karyotype of 46,XX, r(22) (p11.2;q13) [Figure 2]. Because of technical reasons, we could not do FISH for determining the size of deletion. For each family member studied, including proband, 20 metaphases was analyzed. The father (40 years) and mother (35 years) of the proband were healthy. The mother had two pregnancies that resulted in the delivery of healthy child. After cytogenetic analysis of the proband, cytogenetic analyses of both the parents and the sister of the proband were also done. Both mother and father showed normal female karyotpe (46,XX) and male karyotype (46,XY), respectively. The karyotype of her sister was 46,XX. So, this chromosome alteration of the proband was considered as ‘*de novo*’.

## Discussion

Due to its rare cytogenetic finding, the exact incidence of ring chromosome 22 is unknown. Its most consistent clinical findings are overall developmental delay with severe speech disability, growth retardation with frequently associated microcephaly, hypotonia, and dysmorphic traits such as epicanthus, mental retardation, normally placed but large and dysplastic ears, long face, thick eyebrows, 2–3 toe syndactyly, long eyelashes with full eyebrows, and occasionally high arched palate, dental malocclusion, and mild hypertelorism.[1,2] Second and third toe syndactyly, unsteady gait, hyperactivity, aggressive behavior, autistic disorders, and seizures or abnormal EEG1 have also been reported.[3] The other clinical features, dysmorphic traits, anomalies of the extremities, and autistic and behavioral disorders are inconsistent and shared by both ring chromosome 22 and 22qter deletions.

We report a case with ring chromosome 22, who was affected by hypotonia, profound mental retardation, absence of speech, dysmorphic features, long face, thick eyebrows, epicanthic folds, long eyelashes with full eyebrows, and autistic-like behavioral disorders. Magnetic resonance imaging of the brain showed dysmyelini and the patient showed significant behavioral changes and her speech was meaningless. Although the physical features observed in this case can be seen in patients with ring chromosome 22, they are inconsistent with the physical features observed previously in such cases. Our patient had many of the findings previously described in the literature. But growth retardation, occasionally high arched palate, dental malocclusion, mild hypertelorism, poor coordination, syndactyly between toes 2 and 3, and seizures or abnormal EEG have not been reported in our patient. Phenotypic differences in our case might be due to a different deletion size. The recently delineated 22q13.3 deletion syndrome shows some overlapping clinical features with r(22) cases. Among these clinical features, a global developmental delay, particularly severe in the area of expressive language, hypotonia, minor dysmorphic featurs including dolichocephaly, epicanthic folds, syndactyly between toes 2 and 3, and behaviors that overlap with those in the autistic spectrum disorder must be mentioned.[7] For all cases, either 22q13 deletion syndrome cases or r(22) cases, the different clinical features among these individuals may be the result of the different extent and mechanisms of the deletions. Less frequently observed or more severe features might be the consequence of larger deletions. It means that variation in the size of ring chromosomes may contribute to a wide phenotypic spectrum of r(22).

In summary, we reviewed the reports of patients with ring chromosome 22 in the medical literature and compared them with our patient. Our report further delineates the major and minor features seen in this rare cytogenetic syndrome. Our observations of phenotypic differences (and similarities) among subjects with ring chromosome 22 and potential genetic mechanisms that may account for the syndromic features may stimulate efforts to understand the variable clinical presentation of ring chromosome 22 and other ring chromosome syndromes. The wide range of manifestations observed in patients with this cytogenetic alteration is probably due to size differences in the deleted region.
